# Changing Perspective in Dual Disorders: Substance Use, Personality Disorder, and Psychosis

**DOI:** 10.1192/j.eurpsy.2023.1093

**Published:** 2023-07-19

**Authors:** H. Becerra Darriba

**Affiliations:** Psychiatry, Osasunbidea, Tudela, Spain

## Abstract

**Introduction:**

Dual disorders constitute a clinical entity with increasing current prevalence (Köck *et al*. Front Psychiatry 2022; 24 13). There is frequent comorbidity between psychotic spectrum disorders and substance use disorders, which hinders both psychopathological stability and the approach to addictive behaviors (Fleury *et al*. Adm Policy Ment Health 2022; 20).

**Objectives:**

The aim of this study is to describe the clinical and sociodemographic characteristics of the consumption pattern of patients diagnosed with psychosis in outpatient follow-up.

**Methods:**

A cross-sectional study was designed with 42 users treated at the mental health center between 2019 and 2021, aged between 18 and 65 years, who had consumed alcohol, cannabis, and/or stimulants (amphetamines or cocaine), with a diagnosis of a comorbid psychotic spectrum disorder for over 3 years. A descriptive analysis of the prevalence of consumption of each predominant substance was carried out, as well as the sociodemographic and clinical characteristics were collected through a semi-structured interview. Statistical analyzes were performed using SPSS v23.0 (significance p<0.05).

**Results:**

The predominant user profile was a man (85.7%), with a mean age of around 29 years, single (83.3%), with family support (52.4%), resident in rural areas (92.8%), with unqualified employment (57.1%) and primary studies (60%). Cannabis was the predominant substance (80.9%), followed by amphetamines (71.4%), with polydrug use of both in 78.6% of cases. A significant association was found between this combined use of substances, the relapse rate and the presence of comorbid personality disorder.

**Conclusions:**

The paradigm of substance use in psychotic disorders has evolved towards comorbidity with polydrug use and confluence with personality disorders.

**Disclosure of Interest:**

None Declared

